# The effect of educational attainment on birthrate in Japan: an analysis using the census and the vital statistics from 2000 to 2020

**DOI:** 10.1186/s12884-024-06382-6

**Published:** 2024-03-14

**Authors:** Tasuku Okui

**Affiliations:** Medical Information Center, Kyushu University Hospital, Maidashi3-1-1 Higashi-ku, Fukuoka city, Fukuoka prefecture 812-8582 Japan

**Keywords:** Birth rate, Educational attainment, Japan, Vital statistics

## Abstract

**Background:**

In Japan, difference in birth rates depending on educational attainment has not been investigated. This study aimed to reveal birth rates in Japan depending on the highest level of educational attainment and their trends over the years using nationwide government statistics data.

**Methods:**

Individual-level data from Vital Statistics and the Census from 2000, 2010, and 2020 were used for birth and population data, respectively. Data linkage was conducted for males and females in the Census and fathers and mothers in the Vital Statistics using information about gender, household, nationality, marital status, birth year, birth month, prefecture, and municipality for individuals. The birth rate was calculated by gender, a five-year age group, the highest level of educational attainment achieved, and year. In addition, the slope index of inequality (SII) and relative index of inequality (RII) were calculated to evaluate the degree of inequality in birth rates, depending on the educational attainment.

**Results:**

Birth rates were higher in persons with lower educational attainment compared to those with a higher educational attainment among males and females in their twenties, while they tended to be higher in persons with higher educational attainment among those in their thirties and forties. Additionally, an increase in the birth rate from 2000 to 2020 was the largest in university graduates among males aged 25–49 years and women aged 30–49 years, and a decrease in the birth rate was the smallest in university graduates among males and females aged 20–24 years. As a result, SII and RII increased from 2000 to 2020 among males and females in their thirties and forties.

**Conclusions:**

In conclusion, persons with higher educational attainment tended to have a relatively favorable trend in the birth rate compared with persons with lower educational attainment in recent decades. It suggested that enhanced administrative support for individuals with lower educational attainment or lower socioeconomic status may be required to ameliorate the declining birth rate in Japan.

**Supplementary Information:**

The online version contains supplementary material available at 10.1186/s12884-024-06382-6.

## Background

Fertility decline has been observed in many developed countries in recent decades [1, 2] and is also predicted in the twenty-first century globally [3]. Japan is famous for its declining birth rate and aging population. The number of births continues to decrease over decades, and the proportion of elderly persons continues to increase. The number of births decreased from 1,190,547 in 2000 to 840,835 in 2020, with the birth rate decreasing from 9.5 (per 1,000 persons) in 2000 to 6.8 in 2020 [4]. In addition, the number of births declined during the coronavirus disease 2019 pandemic [5]. A declining birth rate is one of the most important social problems in Japan, and this decline will result in a decrease in the labor force and economic power in Japan. It will make it difficult to maintain current social services. Moreover, the decreasing younger generations need to support an increasing number of elderly people, which will reduce the quality of life of younger generations. Birth rate differs depending on the social characteristics of individuals, and several studies investigating an association between social characteristics and fertility have been conducted [6–8]. It is important to assess the current status or causes of the declining birth rate in Japan and discuss countermeasures to the problem. Identifying a social group with relatively low birth rate will be significant for determining a target population of administrative countermeasures for declining birth rates.

Educational level is a major factor that can affect fertility among persons and countries, and the relationship between educational level and fertility varies, depending on regions or countries [9–13]. Some studies showed that the total fertility rate negatively correlates with female education [10, 13]. In addition, a study in Bangladesh showed that female educational attainment has an effect on the decline of fertility desires [14]. In contrast, a study in China showed that a female’s education increased the number of children for women [15], and a positive association between fertility and male educational level was shown in Finland [16]. In Japan, some studies have investigated educational disparities in marital status and the number of children. One study showed that the difference in proportion of married persons depending on educational level attainment grew from 2010 to 2019 [17]. In addition, another study investigated the number of children among persons aged 40–49 years and showed university graduates tended to have children than those who did not graduate university among males [8]. In contrast, there are no studies that investigated the birth rate (number of births per population) depending on educational attainment using national data in Japan. It is important to investigate birth rate for each age group depending on educational attainment because the degree of educational difference in the birth rate can vary depending on the age group. In addition, while it has not been surveyed, the trend of the birth rate might be different by educational attainment for each age group. Moreover, analysis using the Census and Vital Statistics for this purpose could reveal results that represent trends all over Japan.

In this study, we investigated a five-year age group- and gender-specific birth rate depending on educational attainment using nationwide government statistics data in Japan.

## Methods

### Census and vital statistics data

Individual-level data from Vital Statistics and the Census were used for birth data and population data, respectively. The data used in this study were obtained from the Ministry of Internal Affairs and Communications and the Ministry of Health, Labour and Welfare in Japan on the basis of the Statistics Act with the permission of those ministries. The birth data from the Vital Statistics used in this study was from a survey conducted every year, while the Census is conducted every five years in Japan. Data from 2000, 2010, and 2020 were used in the analysis because the Census surveys regarding educational attainment are conducted every ten years. We grouped the educational level into less than high school graduates, high school graduates, junior college or technical college graduates, university or graduate school graduates, persons who were currently enrolled in school, and persons whose educational attainment were unknown. The birth rate of the four types of educational attainment were compared in the analysis. Persons who graduated from university or graduate school were written as university graduates in this study. Number of categories for nationalities were finer for the Census compared with the Vital Statistics in 2010 and 2020, and the categories of the Census were reclassified in order to match those of the Vital Statistics. In addition, only birth month data which were grouped by 3 months were available in the Census in 2010 and 2020. Therefore, the birth month data of fathers and mothers in the Vital Statistics were also grouped by 3 months for data linkage. Birth month data of each individual grouped by 3 months were also used for the Census and the Vital Statistics in 2000 in order to equalize the analysis method with the other years. Moreover, data on birth year were not available for the Census data in 2010 and 2020 and were calculated from the age and birth month of each individual.

Data linkage.

Data linkage was conducted between the Census and Vital Statistics. Unique identification for individuals or personal names was not available in the data whereas information on gender, birth year, birth month, nationality, prefecture, and municipality were available for individuals from the Census. In addition, the birth year and month, nationality, prefecture, and municipality for the fathers and mothers were available from the Vital Statistics. Therefore, deterministic data linkage was conducted between men and women in the Census and fathers and mothers in the Vital Statistics data using the common information. However, too many candidates of fathers and mothers existed for one birth data in this case. Therefore, we set additional restrictions for the matching for births who were born with a marriage [18]. Marital status and household information for individuals can be known from the Census, and whether an infant was born within or without a marriage can be known from the birth data. Therefore, only males and females who were living in a same household and were in a marital relationship in the Census were matched with birth data of infants born with a marriage. Males and females who were in a marital relationship were identified based on marital status and family relationship with the head of a household. According to a survey investigating the proportion of infants aged six months old who were living with their father, approximately 99% of infants born with a marriage were living with their father in Japan in 2001 [19], and the proportion was approximately 98% in 2010 [20]. Therefore, the majority of married couples were living in a same household at the time of childbirth in Japan. Regarding out-of-wedlock births, those were matched with unmarried women. Only one-to-one matched pairs between the Vital Statistics and the Census were used for calculating birth rate.

**Fig. 1:**
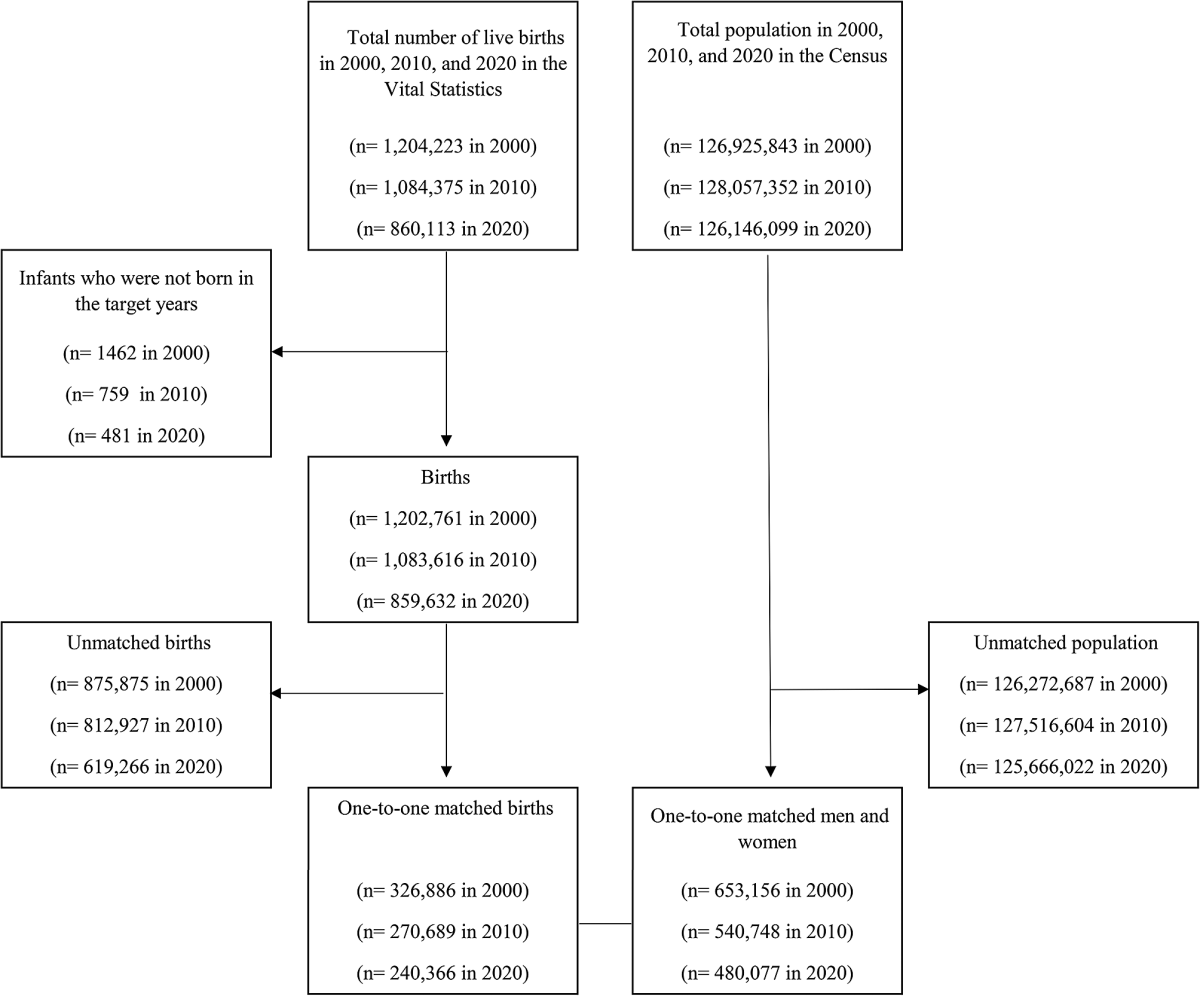
Flowchart of data linkage

Figure 1 shows the flowchart of the data linkage. Parents for 837,941 births in the Vital Statistics were one-to-one matched with 1,673,981 men and women in the Census.

### Statistical analysis

Birth rate was calculated by gender, a five-year age group (15–19 years, 20–24 years, 25–29 years, 30–34 years, 35–39 years, 40–44 years, and 45–49 years), educational attainment, and year. To estimate the birth rate in Japan, adding weight to the matched birth data was required because one-to-one matched birth data were only a part of all birth data and the probability of matching differs depending on birth characteristics. Therefore, the estimated probability of being matched with the Census data for each birth data was calculated by logistic regression analysis using all birth data. Then, an inverse probability of being matched with the Census data was used as a weight for each of the matched birth data to calculate the number of births [21]. The mechanism is same as the inverse probability weighting estimation [22, 23], and it corrects the difference in probability of being matched depending on birth characteristics. In addition, the weight was calibrated to ensure that the sum of the weights became equal to the number of all births in Japan. In the logistic regression analysis, the status of being matched was used as an outcome, and maternal characteristics (combination of maternal age group and nationality), paternal characteristics (combination of paternal age group and nationality), and prefecture were used as explanatory variables for each year as those kinds of information were used in data linkage. In addition, one category of the paternal characteristics was assigned to out-of-wedlock births in order to incorporate them in the analysis. The number of births by age group, educational attainment, gender, and year were estimated by adding up the weights of the matched individuals.

Moreover, to evaluate degree of disparity in birth rate depending on educational attainment, the slope index of inequality (SII) and relative index of inequality (RII) were calculated. These indicators are often used for evaluating health inequalities depending on socioeconomic status [24–26]. The cumulative proportion of the population with an educational attainment or lower was calculated in the order of the educational attainment, and the midpoint of the cumulative proportion was calculated for each of the educational attainment. For example, if the proportion of population for less than high school graduates, high school graduates, junior college or technical college graduates, and university graduates were 0.2, 0.2, 0.4, and 0.4, respectively, the midpoints of the cumulative proportion of population become 0.1, 0.3, 0.6, and 0.8, respectively. Then, a linear regression model was used in order to calculate SII using the birth rate of each educational attainment as an outcome and the midpoints of the cumulative proportion of population as an explanatory variable. Log-transformed birth rate was used as an outcome for RII [25]. A detailed method of calculating these indicators is written in other studies [27, 28]. The SII can be interpreted as the difference in birth rate between the highest and lowest levels of educational attainment, and the RII can be interpreted as the birth rate ratio. The analysis was carried out for each age group, gender, and year. SII and RII for the age group from 15 to 19 years was not calculated because university graduates do not exist in this age group.

Furthermore, in addition to the birth rate among the general population, the birth rate among married persons was calculated. In the calculation of birth rate among married persons, birth data for infants born without a marriage was not used. All statistical analyses were performed using the statistical software R (version 4.6.1) [29]. Statistics shown in this study were created by the author using data that were provided from the Ministries in Japan and are different from those that were published by the Ministries.

## Results

Table 1 shows the proportion of persons with each educational attainment level by gender, year, and age group. The proportion of high school graduates and less than high school graduates showed a decreasing trend in many age groups over the years, both among males and females. For example, the proportion of high school graduates decreased from 39.0% in 2000 to 29.1% in 2020 among men aged 20–24 years, and from 34.9% in 2000 to 24.1% in 2020 among women. However, the proportion of university graduates showed increasing trend over the years regardless of gender among persons aged 25–49 years.

**Table 1:** Proportion of persons with each educational attainment by gender, and year, and age group

Educational attainment, year, and gender	Age group						
	15–19	20–24	25–29	30–34	35–39	40–44	45–49
	%	%	%	%	%	%	%
Men							
2000							
Less than high school	5.8	6.8	7.7	7.8	7.0	9.0	15.8
High school	10.5	39.0	42.5	44.4	45.9	45.1	47.3
Technical school or junior college	0.0	10.5	13.1	10.9	8.9	7.0	5.1
University or more	0.0	10.8	29.1	31.5	34.1	35.4	28.2
Currently enrolled in school	83.7	30.2	2.2	0.6	0.2	0.1	0.0
Unknown	0.0	2.8	5.4	4.7	4.0	3.5	3.6
2010							
Less than high school	3.4	4.4	5.3	5.3	5.8	6.1	5.6
High school	8.9	30.4	30.7	33.9	37.0	39.0	40.9
Technical school or junior college	0.0	8.6	11.1	12.1	12.4	10.1	8.2
University or more	0.0	12.5	31.7	31.1	28.8	29.8	32.1
Currently enrolled in school	87.6	35.2	2.4	0.7	0.3	0.2	0.1
Unknown	0.0	8.9	18.8	17.0	15.6	14.9	13.0
2020							
Less than high school	1.7	2.6	3.3	4.1	5.3	5.3	6.0
High school	9.4	29.1	26.3	27.3	29.3	32.7	36.0
Technical school or junior college	0.0	7.0	9.0	10.1	11.1	11.7	11.8
University or more	0.0	14.3	37.6	38.2	35.4	32.9	29.8
Currently enrolled in school	88.9	36.6	2.2	0.7	0.3	0.2	0.1
Unknown	0.0	10.4	21.6	19.6	18.7	17.2	16.2
Women							
2000							
Less than high school	3.8	4.8	5.4	5.3	4.8	6.9	14.0
High school	9.1	34.9	40.3	46.9	50.7	51.7	56.3
Technical school or junior college	0.0	27.4	33.1	30.3	28.6	25.8	17.7
University or more	0.0	9.0	16.1	13.5	12.6	12.7	8.9
Currently enrolled in school	87.1	21.9	1.3	0.5	0.2	0.1	0.0
Unknown	0.0	2.0	3.8	3.5	3.1	2.9	3.1
2010							
Less than high school	2.9	4.3	4.0	3.3	3.7	3.8	3.6
High school	7.3	28.1	28.2	30.4	35.7	41.8	45.7
Technical school or junior college	0.0	19.2	24.8	30.0	31.1	28.5	27.2
University or more	0.0	12.4	25.9	21.5	15.9	13.1	12.3
Currently enrolled in school	89.7	28.2	1.5	0.6	0.3	0.3	0.2
Unknown	0.0	7.9	15.8	14.2	13.2	12.6	11.1
2020							
Less than high school	1.2	2.0	2.9	3.7	3.7	3.1	3.7
High school	6.6	24.1	22.2	25.0	27.5	29.8	35.2
Technical school or junior college	0.0	15.4	19.2	21.4	24.4	29.2	30.2
University or more	0.0	15.9	35.7	32.0	27.7	22.5	16.7
Currently enrolled in school	92.1	33.2	1.5	0.6	0.3	0.2	0.2
Unknown	0.0	9.4	18.6	17.2	16.4	15.1	14.0

Table 2 shows the estimated number of births per 1,000 persons by gender, year, and educational attainment for each age group. The birth rate tended to be higher in persons with lower educational attainment compared to those with a higher educational attainment among males and females in their twenties, while the birth rate tended to be higher in persons with a higher educational attainment among those in their thirties and forties. In addition, an increase in the birth rate from 2000 to 2020 tended to be the largest in university graduates among males aged 25–49 years and women aged 30–49 years, and a decrease in the birth rate was the smallest in university graduates among males and females aged 20–24 years. For example, among men aged 30–34 years, the birth rate increased from 82.3 in 2000 to 96.7 in 2020 for university graduates, and the degree of increase in the value was the largest among the educational attainment groups. Moreover, the birth rate decreased from 10.1 in 2000 to 7.3 in 2020 for university graduates among men aged 20–24 years, and the degree of decrease in the value was the lowest among the educational attainment groups.

**Table 2:** Birth rate (estimated number of births per 1,000 persons) by gender, year, and educational attainment for each age group

Educational attainment, year, and gender	Age group						
	15–19	20–24	25–29	30–34	35–39	40–44	45–49
Men							
2000							
Less than high school	15.6	84.5	96.9	79.6	45.8	16.3	5.3
High school	7.6	42.9	93.3	97.3	54.7	16.5	5.1
Technical school or junior college	-	29.5	81.1	94.3	58.4	18.5	5.9
University or more	-	10.1	47.0	82.3	53.3	17.2	5.4
2010							
Less than high school	13.7	80.6	109.6	85.0	45.2	16.7	8.2
High school	6.3	41.1	94.4	93.9	49.3	17.2	7.2
Technical school or junior college	-	31.6	99.9	105.3	56.0	19.6	9.0
University or more	-	13.3	61.9	94.6	56.2	19.6	8.7
2020							
Less than high school	11.1	76.0	94.7	78.3	49.6	18.6	8.0
High school	3.5	27.6	77.2	84.8	51.7	17.1	7.0
Technical school or junior college	-	22.1	82.6	105.8	63.5	20.8	8.4
University or more	-	7.3	52.3	96.7	62.0	21.7	9.5
Women							
2000							
Less than high school	62.0	111.2	98.3	77.5	37.3	5.8	0.2
High school	20.3	71.3	119.9	99.2	39.5	6.2	0.1
Technical school or junior college	-	29.7	87.7	92.1	42.6	6.9	0.2
University or more	-	9.8	45.6	75.3	43.3	7.5	0.2
2010							
Less than high school	45.7	110.6	112.9	77.5	45.3	14.1	0.9
High school	18.8	66.7	115.0	96.4	49.6	11.4	0.3
Technical school or junior college	-	31.9	98.8	101.1	55.1	13.2	0.4
University or more	-	10.4	53.9	89.8	59.7	15.1	0.7
2020							
Less than high school	31.7	100.4	108.1	82.6	47.2	14.5	0.5
High school	10.8	46.7	97.9	92.1	55.1	14.3	0.4
Technical school or junior college	0.0	24.6	93.9	107.7	65.6	16.2	0.6
University or more	-	6.6	50.7	97.2	69.0	21.2	1.0

Supplementary Table 1 shows the estimated number of births per 1,000 married persons by gender, year, and educational attainment for each age group. University graduates typically had the lowest birth rates among men aged 20–44 years. For example, the birth rate for university graduates aged 30–34 years was 139.7 per 1,000 men in 2000, which was smaller than those of other educational attainments. In contrast, the birth rate for university graduates tended to be the lowest only among persons aged 20–29 years for women. In addition, the birth rate was often higher in university graduates or technical school or junior college graduates compared with high school graduates or less than high school graduates among women aged 30–49 years. Furthermore, regardless of educational attainment, the birth rate increased among married persons aged 25–49 years from 2000 to 2020 for males and females.

Table 3 shows the results of the inequality indexes for birth rate, depending on educational attainment by age group, gender, and year. An SII value larger than 0 or a RII value larger 1 indicates that the birth rate increases with an increase in educational attainment and an educational disparity in the birth rate exists. The values of SII and RII were below 0 and 1, respectively, among persons aged 20–29 years, indicating that the birth rate decreased with an increase in educational attainment. In contrast, the values of SII and RII tended to be above 0 and 1, respectively, among individuals aged 30–49 years. This indicated that the birth rate increased with an increase in educational attainment, and an inequality by educational attainment existed among persons aged 30–49 years. Moreover, SII and RII values increased from 2000 to 2020 among males and females aged 30–49 years. For example, estimates of SII for men aged 30–34 years increased from 2.8 in 2000 to 29.4 in 2020, and estimates of RII increased from 1.04 in 2000 to 1.39 in 2020. This indicates that inequality by educational attainment increased from 2000 to 2020 among males and females aged 30–49 years.

**Table 3:** Results of inequality indexes for birth rate depending on educational attainment

	Age group					
	20–24	25–29	30–34	35–39	40–44	45–49
Year and gender	Estimates (95%CI)	Estimates (95%CI)	Estimates (95%CI)	Estimates (95%CI)	Estimates (95%CI)	Estimates (95%CI)
SII*						
Men						
2000	-78.9 (−135.3, −22.5)	-58.6 (−144.6, 27.3)	2.8 (-73.8, 79.5)	10.4 (-24.5, 45.2)	1.9 (-4.8, 8.5)	0.4 (-2.5, 3.3)
2010	-72.7 (−135.0, −10.4)	-54.2 (−149.4, 41.0)	15.2 (-43.5, 73.9)	15.0 (1.9, 28.2)	4.2 (-0.4, 8.8)	1.3 (-4.3, 7.0)
2020	-74.0 (−167.1, 19.0)	-51.2 (−124.8, 22.3)	29.4 (-47.6, 106.3)	19.2 (-12.0, 50.5)	5.2 (-5.7, 16.0)	2.2 (-4.0, 8.3)
Women						
2000	-110.1 (−149.6, −70.5)	-64.0 (−205.5, 77.6)	-4.1 (−89.9, 81.7)	6.8 (4.3, 9.3)	1.9 (1.2, 2.6)	0.0 (-0.3, 0.4)
2010	-112.2 (−166.7, −57.6)	-71.9 (−169.8, 25.9)	12.3 (-60.8, 85.4)	16.0 (13.7, 18.3)	1.7 (-9.1, 12.5)	-0.1 (−2.1, 1.8)
2020	-103.3 (−210.8, 4.2)	-72.2 (−148.3, 4.0)	20.4 (-48.8, 89.5)	27.1 (6.4, 47.7)	7.8 (-2.3, 18.0)	0.5 (-0.4, 1.4)
RII*						
Men						
2000	0.12 (0.01, 1.24)	0.43 (0.10, 1.83)	1.04 (0.44, 2.47)	1.23 (0.63, 2.39)	1.12 (0.77, 1.63)	1.07 (0.63, 1.82)
2010	0.15 (0.03, 0.87)	0.52 (0.16, 1.74)	1.18 (0.64, 2.16)	1.34 (1.04, 1.74)	1.26 (0.98, 1.61)	1.18 (0.58, 2.39)
2020	0.09 (0.01, 0.92)	0.48 (0.16, 1.42)	1.39 (0.62, 3.13)	1.41 (0.82, 2.42)	1.30 (0.72, 2.33)	1.29 (0.59, 2.81)
Women						
2000	0.08 (0.01, 0.41)	0.42 (0.07, 2.71)	0.95 (0.35, 2.58)	1.19 (1.11, 1.27)	1.34 (1.23, 1.45)	1.18 (0.14, 9.76)
2010	0.07 (0.01, 0.38)	0.41 (0.11, 1.61)	1.16 (0.51, 2.62)	1.36 (1.27, 1.46)	1.13 (0.49, 2.61)	0.86 (0.02, 34.64)
2020	0.05 (0.01, 0.28)	0.38 (0.12, 1.26)	1.25 (0.61, 2.55)	1.59 (1.06, 2.40)	1.57 (0.92, 2.69)	2.14 (0.51, 9.02)

CI, confidence interval; SII, slope index of inequality; RII, relative index of inequality

*The linear regression model was used to calculate RII and SII.

## Discussion

This study investigated trends in the number of newborns per population by educational attainment in Japan. To the best of our knowledge, this is the first study to demonstrate difference in birth rate depending on educational attainments in Japan using national birth data and investigate the change in the difference over the years. The results demonstrate that the relationship between educational attainment and birth rate differed depending on age and the birth rate was rather higher in those with lower educational levels in their twenties. However, the results of the inequality indicators showed that individuals with higher educational attainment tended to have a relatively favorable trend in the birth rate compared to those with lower educational attainment over the past decades. Possible causes and the implications of the results are discussed below.

Among persons aged 20–29 years old, the birth rate was relatively lower in university graduates among males and females. The proportion of married persons is higher in persons with lower education levels compared with the higher educated persons among persons in their twenties in Japan [17], and an opposite trend was observed in persons in their thirties or older. It is known that a delay in marriage occurs among persons with higher educational attainment among males in Japan [30]. Therefore, it is considered that the difference in the proportion of married persons largely affects the difference in the birth rate because the proportion of infants who were born without a marriage is low in Japan [4]. In contrast, the birth rate for university graduates tended to be the lowest among married men aged 20–44 years and among married women aged 20–29 years. A study in Japan indicated that married women with university degrees tend not to have a child compared to those with lower educational attainment [31]. Additionally, the average number of children tends to be higher in men aged 45–49 years with lower educational attainment in Japan [32]. Therefore, it is considered that the result that persons with lower educational attainment had a lower birth rate among men aged 30–49 years was mainly caused by the lower rate of married persons.

Furthermore, inequality in the birth rate, depending on educational attainment, increased among persons in their thirties and forties in both males and females from 2000 to 2020. An increase in disparity in marriage, depending on educational attainment, is a possible causation for the phenomenon. According to the Japanese National Fertility Survey, the proportion of persons who consider economic factors and educational attainment as a requirement for a marriage partner increased from 2002 to 2021 among never-married persons [32].

With regard to the analysis method, SII and RII were used to show an inequality in birth rate depending on educational attainment in this study. Although SII and RII are often used to compare disparities depending on different kinds of socioeconomic status, these methods are primarily used to show an inequality of outcomes depending on one socioeconomic status. We could demonstrate whether the birth rate showed an increasing or decreasing trend with an increase in educational attainment for each age group by showing SIIs and RIIs in this study. It was also possible to show whether inequality depending on educational attainment increased or decreased over the years by using inequality indexes.

As implied in this study, the results showed that persons with a higher educational attainment showed a relatively favorable trend in the birth rate, compared with persons with a lower educational attainment in recent decades, and this trend might continue in the future. The findings suggest that administrative support of marriage for individuals with lower educational attainment may be necessary in males and females to ameliorate the declining birth rate in Japan. In contrast, the birth rate among married persons tends to be lower in persons with university degrees among men, and it is important to scrutinize the reason. Therefore, it is considered that an improvement in the disparity depending on educational attainment or support for individuals with lower educational level might not be sufficient to resolve the declining birth rate issue in Japan.

There are some limitations to this study. First, the Census and the Vital Statistics data in this study needed to be merged; therefore, some mismatches may have occurred in the data linkage, and some birth data could not be merged with the Census data. For example, if parents changed municipality between the birthday of an infant and the survey date of the Census, the birth data could not be merged with the Census data. In addition, there might exist some couples who divorced early after childbirth, and it is possible that data on those couples could not be correctly matched with the birth data. Moreover, as it is a representative indicator of socioeconomic status, we focused on educational attainment in this study. In contrast, income is another major representative indicator of socioeconomic status, although data was unavailable. Income was associated with the number of children for a man in Japan [8], and it is possible that income explains the association between educational attainment and birth rate to some degree. Using different types of data, a study focusing on income as a socioeconomic status will be meaningful in the future. In contrast, this study used the Census and the Vital Statistics in Japan, and the results of this study represent trends in all of Japan.

## Conclusion

We investigated a five-year age group- and gender-specific birth rate depending on educational attainment using nationwide government statistics data in recent decades in Japan. An association between educational attainment and the birth rate differed between persons in their twenties and older age groups. In addition, persons with a higher educational attainment tended to have a relatively favorable birth rate trend in comparison to persons with a lower educational attainment in recent decades. The results suggest that administrative support of marriage for individuals with lower educational attainment or lower socioeconomic status may be required in males and females to ameliorate the declining birth rate in Japan.

### Electronic supplementary material

Below is the link to the electronic supplementary material.

Supplementary Material 1

## Data Availability

The data that support the findings of this study are available from the Ministry of Internal Affairs and Communications and the Ministry of Health, Labour and Welfare in Japan but restrictions apply to the availability of these data, which were used under license for the current study, and so are not publicly available. Data are however available from the author upon reasonable request and permissions of the Ministry of Internal Affairs and Communications and the Ministry of Health, Labour and Welfare in Japan.

## Declarations

This study was approved by the Kyushu University Institutional Review Board for Clinical Research (No. 22221-02). In addition, informed consent was not necessary because we used official statistics data provided by the government on the basis of the Statistics Act in Japan, and it was waived by the Kyushu University Institutional Review Board for Clinical Research.

## Abbreviations

CI: Confidence interval
RII: Relative index of inequality
SII: Slope index of inequality
