# Supplementary material for: The effect of educational attainment on birthrate in Japan: an analysis using the census and the vital statistics from 2000 to 2020
Source: BMC Pregnancy Childbirth. 2024 Mar 14;24:198. doi: 10.1186/s12884-024-06382-6 (PMC10938742; doi:10.1186/s12884-024-06382-6)
Supplement: Supplementary file 1 — Supplementary Material 1 [file 12884_2024_6382_MOESM1_ESM.pdf]

Supplementary table 1: Estimated number of births per 1,000 married persons by gender, year, and educational attainment for each age group.

Educational attainment, year, and gender	Age group						
	15–19	20–24	25–29	30–34	35–39	40–44	45–49
Men							
2000							
Less than high school	516.4	439.0	257.2	160.4	85.9	28.5	8.2
High school	442.6	415.1	271.8	174.7	79.5	22.0	6.4
Technical school or junior college	-	387.7	255.6	166.0	81.1	23.1	7.0
University or more	-	278.3	193.6	139.7	70.4	20.5	6.1
2010							
Less than high school	568.3	469.4	310.9	188.9	92.6	33.1	16.3
High school	481.2	456.0	312.8	190.9	83.9	27.0	10.4
Technical school or junior college	-	428.4	318.0	192.3	87.1	28.1	12.1
University or more	-	358.6	250.6	170.5	80.8	25.6	10.9
2020							
Less than high school	516.8	462.2	295.3	179.1	97.3	36.5	16.1
High school	368.0	400.2	297.5	196.0	96.1	28.6	11.3
Technical school or junior college	-	361.0	291.6	204.2	100.7	30.9	12.3
University or more	-	261.0	218.4	179.8	90.9	29.3	12.5
Women							
2000							
Less than high school	577.2	365.0	198.5	121.9	49.5	7.0	0.1
High school	513.2	371.7	232.2	136.5	47.7	6.9	0.1
Technical school or junior college	-	324.8	206.6	131.1	51.7	7.7	0.1
University or more	-	224.9	149.7	114.5	54.0	8.4	0.3
2010							
Less than high school	613.2	399.7	229.0	136.8	75.8	20.7	0.9
High school	569.2	398.0	263.5	156.0	70.0	15.1	0.4
Technical school or junior college	-	354.3	250.1	156.9	75.0	17.2	0.5
University or more	-	273.2	190.4	149.2	83.4	20.0	0.8
2020							
Less than high school	546.6	369.7	233.7	144.5	78.5	22.4	1.0
High school	516.1	364.0	253.7	161.2	82.4	20.5	0.6
Technical school or junior college	-	307.0	247.3	172.8	90.8	21.8	0.8
University or more	-	198.9	176.0	161.4	95.3	27.6	1.2
